# Association of genetic polymorphisms in vascular endothelial growth factor with susceptibility to coronary artery disease: a meta–analysis

**DOI:** 10.1186/s12881-018-0628-3

**Published:** 2018-07-04

**Authors:** Wen-Qi Ma, Ying Wang, Xi-Qiong Han, Yi Zhu, Nai-Feng Liu

**Affiliations:** Department of Cardiology, Zhongda Hospital, School of Medicine, Southeast University, 87 Dingjiaqiao, Nanjing, 210009 People’s Republic of China

**Keywords:** Vascular endothelial growth factor, Coronary artery disease, Gene polymorphisms, Meta-analysis

## Abstract

**Background:**

Single nucleotide polymorphisms (SNPs) located in the vascular endothelial growth factor (VEGF) gene may be correlated with the susceptibility to coronary artery disease (CAD) – although results have been controversial. The aim of this meta–analysis is to clarify the effects of VEGF –2578A/C (rs699947), −1154G/A (rs1570360), +405C/G (rs2010963), and + 936C/T (rs3025039) polymorphisms on CAD risk.

**Methods:**

Pooled odds ratio (OR) and corresponding 95% confidence intervals (CIs) were calculated to estimate the strength of the association between VEGF gene polymorphisms and CAD risk. Fixed- or random-effects model was used depending on the heterogeneity between studies.

**Results:**

In total, 13 eligible articles containing 29 studies were analysed. The pooled analysis indicated that the VEGF gene polymorphisms of rs699947, rs2010963, and rs3025039 were associated with an increased risk of CAD, whereas no significant associations were observed with the rs1570360 polymorphism. A subgroup analysis stratified by ethnicity revealed that the rs699947 and rs3025039 polymorphisms were associated with CAD risk in Asian populations. In addition, stratification by control source indicated an increased risk of CAD susceptibility with the rs699947 polymorphism for population–based studies of reduced heterogeneity.

**Conclusions:**

In summary, we concluded that the VEGF gene polymorphisms rs699947, rs2010963, and rs3025039 are correlated with an elevated CAD risk.

**Electronic supplementary material:**

The online version of this article (10.1186/s12881-018-0628-3) contains supplementary material, which is available to authorized users.

## Background

Coronary artery disease (CAD) is and will remain the main cause of morbidity and mortality worldwide [1]. CAD is a chronic, progressive, and polygenic disease, and atherosclerosis is appears to be the major pathophysiological process underlying CAD [2]. The roles of endothelial dysfunction and angiogenesis in atherosclerosis development have been widely reported [3, 4]. Endothelial dysfunction, which is frequently triggered by smoking, dyslipidaemia, hypertension, hyperglycaemia, and insulin resistance, may influence the balance between endothelium–dependent vasoconstriction and vasodilatation, the upregulation of cytokines, adhesion molecule expression, leukocyte and monocyte migration, and platelet activation [5, 6]. Moreover, angiogenesis may be related to disturbances in endothelial cell physiology. These complex, associated processes of endothelial dysfunction and angiogenesis require the participation of various growth factors.

Vascular endothelial growth factor (VEGF), an essential component of angiogenesis, has been reported to induce endothelial cell migration and proliferation, enhance vascular permeability, and modulate thrombogenicity [7–9]. The VEGF family includes VEGF–A, VEGF–B, VEGF–C, VEGF–D, VEGF–E, VEGF–F, and placental growth factor, and all these growth factors perform their functions by interacting with high–affinity receptor tyrosine kinases [10]. The VEGF gene is located in chromosome 6p21.3 and contains a 14–kb coding region with eight exons and seven introns [11]. It is expressed in various cell types, such as endothelial cells, vascular smooth muscle cells, macrophages, and several tumour cells. Molecular biology studies have confirmed that VEGF expression is regulated by certain single nucleotide polymorphisms (SNPs), which have tissue- and age-specific expression patterns [12–15]. Furthermore, VEGF gene variability may be of particular interest for many angiogenesis–associated diseases, such as tumours, osteosarcoma, age–related macular degeneration, diabetic retinopathy, and chronic immune–mediated inflammatory diseases [16–20].

Some of these polymorphisms, including VEGF –2578A/C (rs699947), −1154G/A (rs1570360), +405C/G (rs2010963), and + 936C/T (rs3025039), which arise from the vascular expression of different VEGF proteins, have been associated with CAD susceptibility; however, these findings are controversial. For example, Han et al. [21] indicated that two VEGF SNPs (rs2010963 and rs3025039) were associated with CAD susceptibility in a Chinese population. Similarly, Li et al. [22] also reported that the C allele of VEGF (rs699947) may be an important independent risk factor for susceptibility to CAD. However, other studies have drawn opposite conclusions. Biselli et al. [23] suggested a possible protective effect of the rs699947 polymorphism on CAD severity because of a reduced VEGF expression.

The existence of a correlation between VEGF gene SNPs and CAD susceptibility remains controversial and inconclusive. Moreover, no relevant meta–analyses have been published. Consequently, we performed a meta–analysis to investigate the association between VEGF gene polymorphisms and CAD risk.

## Methods

### Literature search strategy

The literature search was conducted by two authors (Ma and Han). PubMed, EMBASE, Web of Science, ScienceDirect, and Cochrane Library were systematically searched. The keywords and terms used for the searches included the following: “vascular endothelial growth factor” OR “VEGF” OR “–2578 A/C” OR “+405C/G” OR “+936C/T” OR “–1154G/A” OR “rs699947” OR “rs3025039” OR “rs2010963” OR “rs1570360”; “genetic polymorphism” OR “mutation” OR “variant” OR “genotype”; and “angina” OR “myocardial infarction” OR “atherosclerosis” OR “acute coronary syndrome” OR “coronary artery disease” OR “coronary heart disease”. Hand–searching of reference lists in relevant articles was also performed. The last search was conducted on October 24, 2017.

### Inclusion and exclusion criteria

Eligible studies in our meta–analysis complied with the following criteria: 1) case–control studies evaluating VEGF gene polymorphisms (rs699947, rs2010963, rs3025039, and rs1570360) and CAD susceptibility; 2) all CAD cases were documented by angiographic evidence showing at least 50% stenosis of one major coronary artery, myocardial infarction (MI), or coronary artery bypass surgery; 3) sufficient published data, such as the total number of cases and controls, distribution of genotypes, and other relevant information; and 4) language was restricted to English. Studies were excluded if they met the following criteria: 1) letters to the editor, abstracts, animal studies, or reviews; 2) data overlapping with previous publications; and 3) studies with unusable or insufficient data.

### Data extraction

Following the Meta–analysis of Observational Studies in Epidemiology (MOOSE) guidelines for reporting meta–analyses of observational studies (Additional file 1: Table S1), data from eligible studies were separately extracted by two authors (Ma and Zhu), and eligibility disagreements were discussed and resolved by a third author (Liu). For each eligible study, data included information regarding the author, year of publication, number of cases and controls, country, ethnicity, genotyping methods, genotype frequency in cases and controls, sources of controls, and Hardy–Weinberg equilibrium (HWE) in controls.

### Quality assessment

The Newcastle–Ottawa Scale (NOS) quality score was applied to assess the quality of each eligible study. Validated quality assessment consisted of three parameters, including selection, comparability, and exposure. NOS scores ranged from 0 and 9 stars. Studies with an NOS score of five or greater were deemed moderate to high quality, whereas studies with an NOS score of less than five were considered low quality.

### Statistics analysis

All calculations and graphs were performed by Review Manager v5.2 (The Cochrane Collaboration, Oxford, UK) and Stata 12.0 (Stata Corporation, College Station, Texas, USA). Pooled odds ratios (ORs) and corresponding 95% confidence intervals (CIs) were selected to estimate the strength of the association. For the VEGF rs6699947 polymorphism, pooled ORs were obtained for dominant (CC + AC vs. AA), recessive (CC vs. AA + AC), homozygous (CC vs. AA), heterozygous (AC vs. AA), and allele (C vs. A) genetic models. Similar genetic models were also used to assess the rs2010963, rs3025039, and rs1570360 polymorphisms. The Cochrane Q–test and index (I ^2^) were used to assess the heterogeneity within studies. A Q–test with *P* < 0.10 indicated significant heterogeneity. I^2^ values of 0–25, 25–50%, and > 50% represented mild, moderate, and high–level heterogeneity, respectively. A fixed– or random–effects model was used to calculate OR and 95% CIs based on the study heterogeneity strength. Subsequently, a subgroup analysis was performed to search for potential sources of heterogeneity. If there was an appropriate number of included studies, subgroup analyses based on ethnicity (Asian and Caucasian populations), control source (population–based and hospital–based controls), and sample size (studies with more than 500 subjects were categorized as “large” and studies with less 500 subjects were categorized as “small”), and the type of CAD were performed to detect the sources of the heterogeneity. A sensitivity analysis was performed to assess the stability of the individual studies. Possible publication bias was assessed using funnel plots and Egger’s linear regression test.

## Results

### Study selection and characteristics

**Fig. 1:**
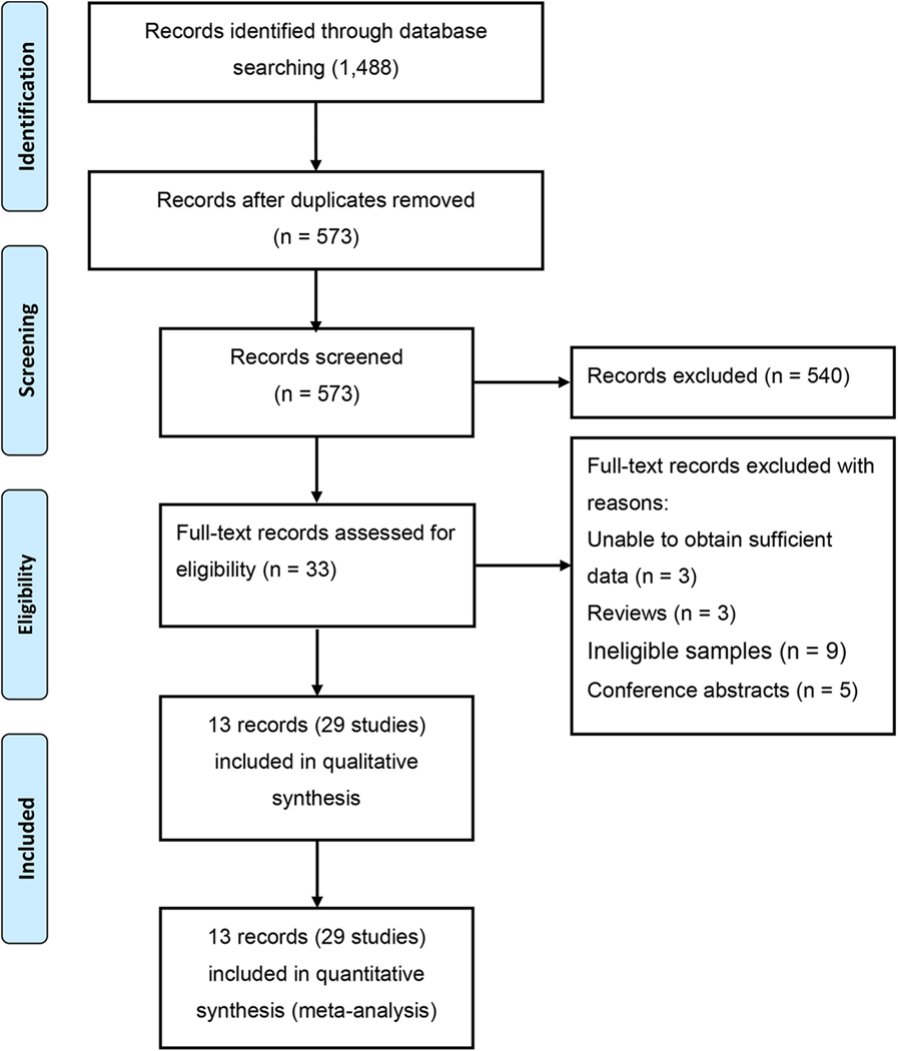
Flow chart of the selection of eligible studies for the meta–analysis

A total of 1488 articles were retrieved following the initial search. Of these publications, 915 were excluded due to duplicate records, 33 articles were determined to be ineligible after the screening of the titles and abstracts, and 20 articles were excluded after reading the full texts because of insufficient data, ineligible samples, or by virtue of being a review or conference abstract. Ultimately, thirteen eligible articles containing 29 studies met our inclusion criteria [21–33]. The study selection process is summarized in Fig. 1. The quality of the included studies was evaluated using the NOS quality score (Additional file 1: Table S2).

The data from the eligible studies are displayed in Table 1. Eight studies containing 2471 cases and 2811 controls found an association between the VEGF rs699947 polymorphism and susceptibility to CAD. Ten studies involving 2303 cases and 2862 controls focused on the relationship between the VEGF rs2010963 polymorphism and susceptibility to CAD. Eight studies of the VEGF rs3025039 polymorphism included 2136 cases and 2477 controls, and three VEGF rs1570360 polymorphism studies included 1227 cases and 1166 controls. The group of controls consisted of healthy volunteers from the community or patients who underwent health examinations in the hospital. Twelve studies were based on Caucasian samples, and seventeen studies used Asian samples. The countries in which the eligible studies were conducted included Brazil, China, Finland, Iran, Slovenia, and the United Kingdom. The distribution of genotypes in the controls was tested in 29 studies and found to be mostly consistent with HWE, except for two studies [31].

**Table 1:** Characteristics of the individual studies included in the meta–analysis

Study	Country	Ethnicity	Disease	Control Source	Genotyping Methods	Sample Size Cases/Controls	Genotype Distribution		P_HWE_	NOS
							Cases	Controls		
rs699947							AA/AC/CC	AA/AC/CC		
Biselli 2008^23^	Brazil	Caucasian	CAD	HB	PCR	175/108	32/96/47	27/51/30	0.569	6
Kangas–Kontio 2009^25^	Finland	Caucasian	MI	HB	TaqMan	188/218	64/87/37	77/101/40	0.498	7
Chen 2011^26^	UK	Caucasian	MI	PB	PCR–RFLP	46/372	10/34/2	100/167/105	0.050	8
Amoli 2012^27^	Iran	Asian	CAD	HB	PCR	50/50	9/26/15	14/27/9	0.520	6
Cui 2013^28^	China	Asian	CAD	HB	PCR	242/253	27/78/137	12/69/172	0.148	6
Gu 2013^29^	China	Asian	CAD	HB	MassARRAY	427/472	30/178/219	31/174/267	0.713	6
Li 2016^22^	China	Asian	CAD	PB	PCR	533/533	180/250/103	217/237/79	0.280	8
Liu 2016^31^	China	Asian	CAD	PB	PCR	810/805	275/381/154	339/345/121	0.03	8
rs2010963							GG/GC/CC	GG/GC/CC		
Petrovic 2007^24^	Slovenia	Caucasian	MI	PB	PCR	143/228	42/76/25	103/104/21	0.470	7
Kangas–Kontio 2009^25^	Finland	Caucasian	MI	HB	TaqMan	186/218	113/61/12	143/67/8	0.966	7
Chen 2011^26^	UK	Caucasian	MI	PB	PCR–RFLP	46/372	26/19/1	174/159/39	0.765	8
Cui 2013^28^	China	Asian	CAD	HB	PCR	242/253	75/102/65	104/114/35	0.675	6
Gu 2013^29^	China	Asian	CAD	HB	MassARRAY	419/468	144/215/60	154/225/89	0.672	6
Douvaras 2013^32^	Greece	Caucasian	MI	HB	PCR–RFLP	102/98	37/49/16	29/55/14	> 0.050	7
Moradzadegan 2015^30^	Iran	Asian	CAD	HB	PCR–RFLP	141/369	43/65/33	85/197/87	0.193	7
Han 2015^21^	China	Asian	CAD	HB	MassARRAY	144/150	69/49/26	86/54/10	0.701	6
Li 2016^22^	China	Asian	CAD	PB	PCR	533/533	50/233/250	71/239/223	0.583	8
Nia 2017^33^	Iran	Asian	CAD	HB	TaqMan	347/173	167/135/45	102/63/8	> 0.050	7
rs3025039							CC/CT/TT	CC/CT/TT		
Biselli 2008^23^	Brazil	Caucasian	CAD	HB	PCR	175/108	133/36/6	83/23/2	0.783	6
Kangas–Kontio 2009^25^	Finland	Caucasian	MI	HB	TaqMan	187/218	140/42/5	155/56/7	0.488	7
Chen 2011^26^	UK	Caucasian	MI	PB	PCR–RFLP	46/372	37/8/1	264/95/13	0.229	8
Cui 2013^28^	China	Asian	CAD	HB	PCR	242/253	133/95/14	159/86/8	0.373	6
Gu 2013^29^	China	Asian	CAD	HB	MassARRAY	430/473	272/142/16	300/159/14	0.194	6
Douvaras 2013^32^	Greece	Caucasian	MI	HB	PCR–RFLP	102/98	68/30/4	69/27/2	> 0.050	7
Han 2015^21^	China	Asian	CAD	HB	MassARRAY	144/150	84/55/5	115/31/4	0.290	6
Liu 2016^31^	China	Asian	CAD	PB	PCR	810/805	472/308/30	617/167/21	0.020	8
rs1570360							GG/GA/AA	GG/GA/AA		
Biselli 2008^23^	Brazil	Caucasian	CAD	HB	PCR	175/108	96/61/18	57/38/13	0.104	6
Cui 2013^28^	China	Asian	CAD	HB	PCR	242/253	151/79/12	172/69/12	0.148	6
Liu 2016^31^	China	Asian	CAD	PB	PCR	810/805	54/370/386	137/456/212	> 0.050	8

Abbreviations: *CAD* coronary artery disease; *MI* myocardial infarction; *PCR* polymerase chain reaction; *PCR–RFLP* polymerase chain reaction-restriction fragment length polymorphism; *HWE* Hardy–Weinberg equilibrium; *PB* population–based; *HB* hospital–based; *NOS* Newcastle–Ottawa quality scale; *UK* the United Kingdom

### Association between the rs699947 polymorphism and susceptibility to CAD

For all studies, the meta–analysis showed an increased risk between the rs699947 polymorphism and CAD susceptibility in the heterozygous genetic model (AC vs. AA: OR = 1.26, 95% CI = 1.10–1.45) with a low between–study heterogeneity. No significant associations were observed in the dominant (CC + AC vs. AA: OR = 1.17, 95% CI: 0.93–1.47), recessive (CC vs. AA + AC: OR = 0.96, 95% CI: 0.71–1.31), homozygous (CC vs. AA: OR = 1.05, 95% CI: 0.71–1.55), and allele (C vs. A: OR = 1.01, 95% CI: 0.83–1.23) genetic models (Table 2; Additional file 1: Figs. S1-S5). Stratification by ethnicity indicated that the rs699947 polymorphism was significantly associated with CAD risk in Asian populations compared to Caucasian populations under the heterozygous genetic model (AC vs. AA: OR = 1.25, 95% CI = 1.07–1.46). In the subgroup analysis stratified by the control source, we observed a significantly increased risk of CAD susceptibility in the dominant genetic model (CC + AC vs. AA: OR = 1.38, 95% CI: 1.19–1.61) within population–based studies with reduced heterogeneity. A similar result was also detected for the heterozygous genetic model. When we conducted a subgroup analysis by sample size, the same significant associations were observed in studies with large sample sizes in dominant and heterozygous genetic models with a low heterogeneity (Table 2).

**Table 2:** Summary of odds ratios (95% CI) in the analysis of the relationship between VEGF gene polymorphisms in rs699947 and coronary artery disease susceptibility

Genetic Model	Overall and Subgroups	N	Test of Association			Test of Heterogeneity	
			OR	95%CI	*P*_*–value*_	*P*_Heterogeneity_	I^2^ (%)
	Overall	8	1.17	0.93,1.47	0.170	0.040	52%
	HWE (yes)	7	1.10	0.83,1.47	0.520	0.050	53%
	PB	3	1.38	1.19,1.61	0.000	0.950	0%
CC+ AC vs. AA	HB	5	0.99	0.65,1.51	0.970	0.040	65%
	Large sample size	3	1.34	1.16,1.56	0.000	0.340	8%
	Small sample size	5	1.06	0.67,1.68	0.800	0.040	61%
	Asians	5	1.11	0.79,1.55	0.550	0.009	71%
	Caucasians	3	1.21	0.89,1.64	0.220	0.620	0%
	Overall	8	0.96	0.71,1.31	0.800	0.000	75%
	HWE (yes)	7	0.90	0.63,1.27	0.550	0.000	74%
	PB	3	0.98	0.53,1.80	0.950	0.003	83%
CC vs. AA+ AC	HB	5	0.87	0.66,1.14	0.320	0.130	45
	Large sample	3	1.13	0.80,1.60	0.480	0.010	78%
	Small sample	5	0.80	0.47,1.37	0.420	0.006	73%
	Asians	5	0.99	0.74,1.48	0.790	0.000	79%
	Caucasians	3	0.65	0.27,1.56	0.340	0.010	78%
	Overall	8	1.26	1.10,1.45	0.001	0.210	27%
	HWE (yes)	7	1.20	1.00,1.44	0.050	0.180	32%
	PB	3	1.36	1.15,1.60	0.000	0.510	0%
AC vs. AA	HB	5	1.05	0.81,1.37	0.710	0.210	32%
	Large sample	3	1.30	1.11,1.52	0.001	0.680	0%
	Small sample	5	1.16	0.88,1.54	0.290	0.080	53%
	Asians	5	1.25	1.07,1.46	0.005	0.150	41%
	Caucasians	3	1.33	0.97,1.83	0.080	0.250	29%
	Overall	8	1.05	0.71,1.55	0.810	0.001	72%
	HWE (yes)	7	0.95	0.59,1.53	0.770	0.001	72%
	PB	3	1.29	0.77,2.17	0.340	0.030	72%
CC vs. AA	HB	5	0.95	0.57,1.60	0.850	0.020	65%
	Large sample	3	1.36	0.99,1.88	0.060	0.110	54%
	Small sample	5	0.82	0.39,1.71	0.600	0.004	74%
	Asians	5	1.12	0.68,1.83	0.660	0.001	79%
	Caucasians	3	0.88	0.41,1.89	0.740	0.070	62%
	Overall	8	1.01	0.83,1.23	0.930	0.000	80%
	HWE (yes)	7	0.96	0.77,1.21	0.750	0.000	77%
	PB	3	1.14	0.90,1.45	0.280	0.020	73%
C vs. A	HB	5	0.95	0.73,1.22	0.680	0.008	71%
	Large sample	3	1.13	0.90,1.43	0.290	0.005	81%
	Small sample	5	0.92	0.67,1.27	0.620	0.005	73%
	Asians	5	1.04	0.79,1.37	0.790	0.000	86%
	Caucasians	3	0.97	0.75,1.26	0.820	0.180	42%

Abbreviations: *N* number of studies; *OR* odds ratio; *CI* confidence interval; *HB* hospital–based; *PB* population–based; *P*_–Value_, *P* value for association; *P*_Heterogeneity_, *P* value for heterogeneity

### Association between the rs2010963 polymorphism and susceptibility to CAD

A significant association between rs2010963 polymorphism and CAD risk was found under the recessive and homozygous genetic models (CC vs. GG + GC: OR = 1.45, 95% CI = 1.03–2.05; CC vs. GG: OR = 1.57, 95% CI = 1.02–2.42) (Table 3; Additional file 1: Figs. S6-S10). Stratification by sample size indicated that the rs2010963 polymorphism was significantly associated with CAD risk for small sample sizes compared to large sample sizes in several genetic models (CC vs. GG + GC: OR = 1.52, 95% CI = 1.01–2.33; CC vs. GG: OR = 2.03, 95% CI = 1.26–3.28; C vs. G: OR = 1.27, 95% CI = 1.03–1.78). No significant associations were observed following a subgroup analysis by ethnicity and control source (Table 3).

**Table 3:** Summary of odds ratios (95% CI) in the analysis of the relationship between VEGF gene polymorphisms in rs2010963 and coronary artery disease susceptibility

Genetic Model	Overall and Subgroups	N	Test of Association			Test of Heterogeneity	
			OR	95%CI	*P*_*–value*_	*P*_*Heterogeneity*_	I^2^ (%)
	Overall	10	1.18	0.94,1.48	0.140	0.002	65%
	PB	3	1.31	0.77,2.25	0.320	0.020	74%
CC + GC vs. GG	HB	7	1.13	0.88,1.44	0.340	0.010	62%
	Large sample size	4	1.11	0.77,1.58	0.580	0.009	74%
	Small sample size	6	1.25	0.92,1.68	0.150	0.030	60%
	Asians	6	1.22	0.93,1.59	0.140	0.010	67%
	Caucasians	4	1.09	0.68,1.76	0.720	0.010	72%
	Overall	10	1.45	1.03,2.05	0.030	0.000	74%
	PB	3	1.26	0.64,2.48	0.500	0.050	67%
CC vs. GG + GC	HB	7	1.56	0.97,2.53	0.070	0.000	79%
	Large sample size	4	1.43	0.74,2.74	0.290	0.000	72%
	Small sample size	6	1.52	1.01,2.33	0.040	0.050	55%
	Asians	6	1.50	0.98,2.31	0.060	0.000	82%
	Caucasians	4	1.35	0.70,2.60	0.370	0.110	51%
	Overall	10	1.18	0.90,1.57	0.240	0.000	76%
	PB	3	1.32	0.88,1.98	0.180	0.130	51%
GC vs. GG	HB	7	1.14	0.79,1.64	0.490	0.000	81%
	Large sample size	4	1.28	0.72,2.28	0.400	0.060	65%
	Small sample size	6	1.14	0.89,1.46	0.290	0.190	32%
	Asians	6	1.25	0.85,1.84	0.260	0.000	83%
	Caucasians	4	1.08	0.72,1.63	0.710	0.060	59%
	Overall	10	1.57	1.02,2.42	0.040	0.000	77%
	PB	3	1.47	0.60,3.63	0.400	0.020	74%
CC vs. GG	HB	7	1.60	0.93,2.77	0.090	0.000	81%
	Large sample size	4	1.24	0.68,2.28	0.480	0.000	71%
	Small sample size	6	2.03	1.26,3.28	0.007	0.070	50%
	Asians	6	1.62	0.94,2.79	0.080	0.000	84%
	Caucasians	4	1.40	0.42,4.61	0.580	0.002	73%
	Overall	10	1.19	0.98,1.44	0.080	0.000	78%
	PB	3	1.15	0.77,1.73	0.500	0.006	81%
C vs. G	HB	7	1.20	0.94,1.53	0.140	0.000	80%
	Large sample size	4	1.10	0.84,1.43	0.490	0.001	814%
	Small sample size	6	1.27	1.03,1.78	0.030	0.010	72%
	Asians	6	1.24	0.98,1.58	0.070	0.000	83%
	Caucasians	4	1.08	0.73,1.60	0.690	0.006	76%

Abbreviations: *N* number of studies; *OR* odds ratio; *CI* confidence interval; *HB* hospital–based; *PB* population–based; *P*_*–Value*_, *P* value for association; *P*_*Heterogeneity*_, *P* value for heterogeneity

### Association between the rs3025039 polymorphism and susceptibility to CAD

Meta–analysis of the rs3025039 polymorphism showed an elevated risk of CAD in the homozygous genetic model (TT vs. CC: OR = 1.55, 95% CI = 1.10–2.17) with a low between–study heterogeneity. No significant associations were observed in the remaining genetic models (TT+ CT vs. CC: OR = 1.26, 95% CI: 0.89–1.79; TT vs. CC + CT: OR = 1.38, 95% CI: 0.98–1.93; CT vs. CC: OR = 1.24, 95% CI: 0.86–1.80; T vs. C: OR = 1.23, 95% CI: 0.93–1.62) (Table 4; Additional file 1: Figs. S11-S15). The subgroup analysis stratified by ethnicity indicated that the rs3025039 polymorphism was significantly associated with CAD in Asian populations compared to Caucasian populations for all genetic models, except for the dominant and recessive genetic models. Stratification by sample size indicated that the rs3025039 polymorphism was significantly associated with CAD for large sample sizes in the homozygous genetic model. No significant associations were observed in the subgroup analysis stratified by the control source (Table 4).

**Table 4:** Summary of odds ratios (95% CI) in the analysis of the relationship between VEGF gene polymorphisms in rs3025039 and coronary artery disease susceptibility

Genetic Model	Overall and Subgroups	N	Test of Association			Test of Heterogeneity	
			OR	95%CI	*P*_*–value*_	*P*_*Heterogeneity*_	I^2^ (%)
	Overall	8	1.26	0.89,1.79	0.200	0.000	84%
	HWE (yes)	7	1.14	0.86,1.50	0.360	0.020	59%
	PB	2	1.24	0.32,4.77	0.750	0.000	91%
TT + CT vs. CC	HB	6	1.17	0.82,1.65	0.390	0.020	63%
	Large sample size	2	1.55	0.67,3.54	0.300	0.000	96%
	Small sample size	6	1.15	0.76,1.75	0.500	0.008	71%
	Asians	4	1.17	0.82,1.65	0.390	0.020	63%
	Caucasians	4	0.91	0.69,1.20	0.40	0.490	0%
	Overall	8	1.38	0.98,1.93	0.060	0.950	0%
	HWE (yes)	7	1.35	0.88,2.05	0.170	0.910	0%
	PB	2	1.34	0.78,2.29	0.290	0.430	0%
TT vs. CC + CT	HB	6	1.40	0.91,2.17	0.130	0.910	0%
	Large sample size	2	1.37	0.88,2.14	0.170	0.790	0%
	Small sample size	6	1.40	0.91,2.17	0.130	0.910	0%
	Asians	4	1.45	0.99,2.12	0.060	0.920	0%
	Caucasians	4	1.14	0.55,2.36	0.730	0.710	0%
	Overall	8	1.24	0.86,1.80	0.250	0.000	84%
	HWE (yes)	7	1.11	0.84,1.47	0.440	0.030	58%
	PB	2	1.27	0.33,4.95	0.730	0.001	91%
CT vs. CC	HB	6	1.18	0.89,1.56	0.250	0.040	58%
	Large sample size	2	1.55	0.64,3.72	0.330	0.000	96%
	Small sample size	6	1.18	0.89,1.56	0.250	0.040	58%
	Asians	4	1.64	1.01,2.69	0.050	0.000	89%
	Caucasians	4	0.89	0.66,1.19	0.410	0.640	0%
	Overall	8	1.55	1.10,2.17	0.010	0.830	0%
	HWE (yes)	7	1.39	0.91,2.13	0.120	0.820	0%
	PB	2	1.67	0.98,2.85	0.060	0.260	21%
TT vs. CC	HB	6	1.47	0.95,2.28	0.080	0.830	0%
	Large sample size	2	1.61	1.03,2.53	0.040	0.410	0%
	Small sample size	6	1.47	0.95,2.28	0.080	0.830	0%
	Asians	4	1.70	1.16,2.50	0.007	0.810	0%
	Caucasians	4	1.10	0.53,2.28	0.810	0.650	0%
	Overall	8	1.23	0.93,1.62	0.140	0.000	80%
	HWE (yes)	7	1.13	0.91,1.42	0.270	0.040	54%
	PB	2	1.17	0.38,3.57	0.780	0.002	90%
T vs. C	HB	6	1.19	0.96,1.48	0.110	0.070	51%
	Large sample size	2	1.43	0.76,2.70	0.270	0.000	95%
	Small sample size	6	1.19	0.96,1.48	0.110	0.070	51%
	Asians	4	1.50	1.06,2.13	0.020	0.000	85%
	Caucasians	4	0.94	0.74,1.21	0.640	0.390	0%

Abbreviations: *N* number of studies; *OR* odds ratio; *CI* confidence interval; *HB* hospital–based; *PB* population–based; *P*_*–Value*_, *P* value for association; *P*_*Heterogeneity*_, *P* value for heterogeneity

### Association between the rs1570360 polymorphism and susceptibility to CAD

No significant associations were observed between the rs1570360 polymorphism and CAD susceptibility in all genetic models (Table 5). Due to the limited number of eligible studies on rs1570360, no further subgroup analysis was performed.

**Table 5:** Summary of odds ratios (95% CI) in the analysis of the relationship between VEGF gene polymorphisms in rs1570360 and coronary artery disease susceptibility

Genetic Model	N	Test of Association			Test of Heterogeneity	
		OR	95%CI	*P*_*–value*_	*P*_*Heterogeneity*_	I^2^ (%)
AA + GA vs. GG	3	1.52	0.77,3.00	0.220	0.000	89%
AA vs. GG + GA	3	1.40	0.62,3.15	0.410	0.004	82%
GA vs. GG	3	1.41	0.91,2.18	0.120	0.040	70%
AA vs. GG	3	1.70	0.50,5.73	0.390	0.000	91%
A vs. G	3	1.32	0.81,2.16	0.260	0.000	90%

Abbreviations: *N* number of studies; *OR* odds ratio; *CI* confidence interval; *P*_*–Value*_, *P* value for association; *P*_*Heterogeneity*_, *P* value for heterogeneity

### Sensitivity analysis

**Fig. 2:**
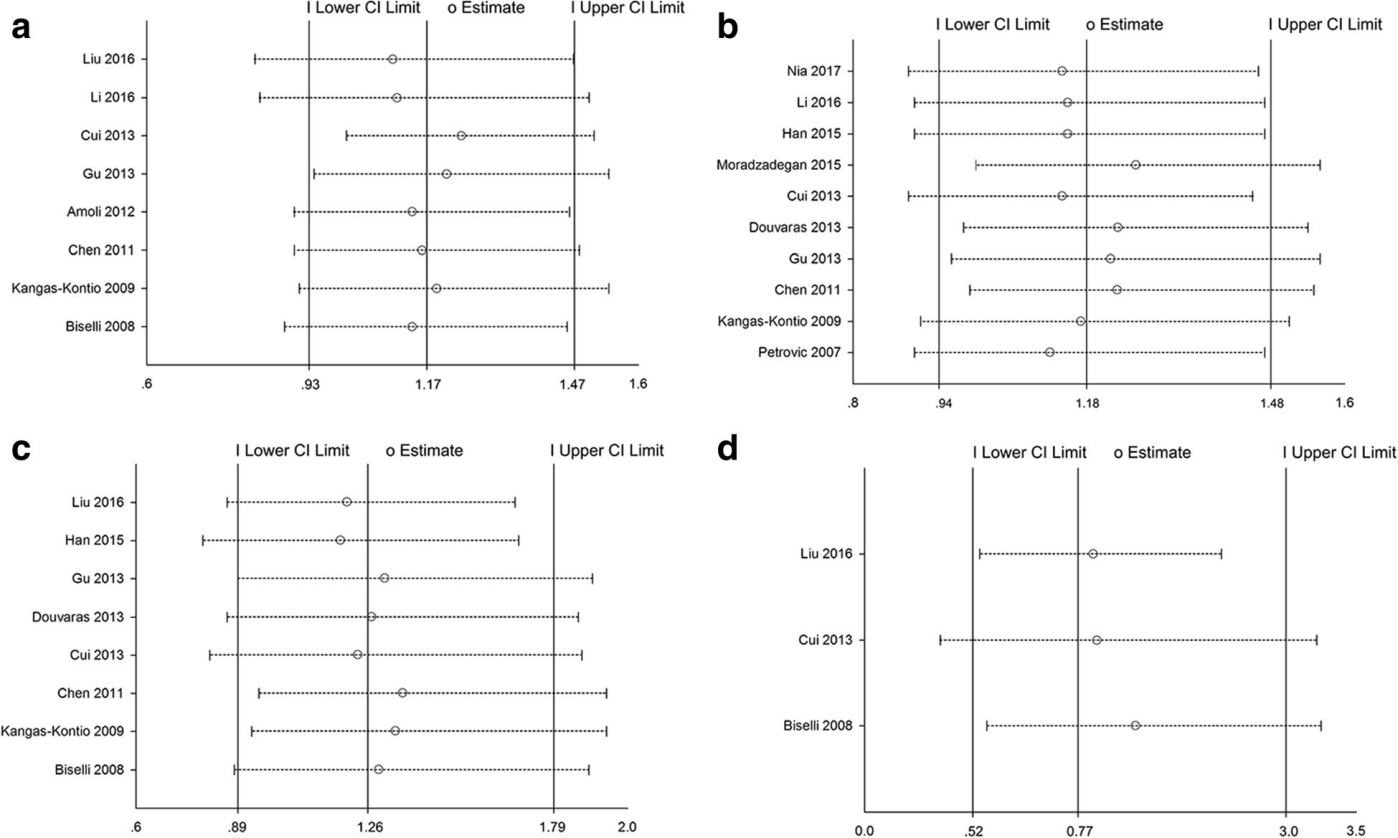
Sensitivity analysis on the association between VEGF gene polymorphisms and susceptibility to CAD. **a** Sensitivity analysis for rs699947 and CAD risk; **b** Sensitivity analysis for rs2010963 and CAD risk; **c** Sensitivity analysis for rs3025039 and CAD risk; (**d**) Sensitivity analysis for rs1570360 and CAD risk. Abbreviation: CI = confidence interval

The influence of each study on the overall meta–analysis was evaluated by excluding each study at a time. The sensitivity analysis confirmed the stability of our meta-analysis and indicated that no individual study significantly affected the pooled result (Fig. 2).

### Publication Bias

**Fig. 3:**
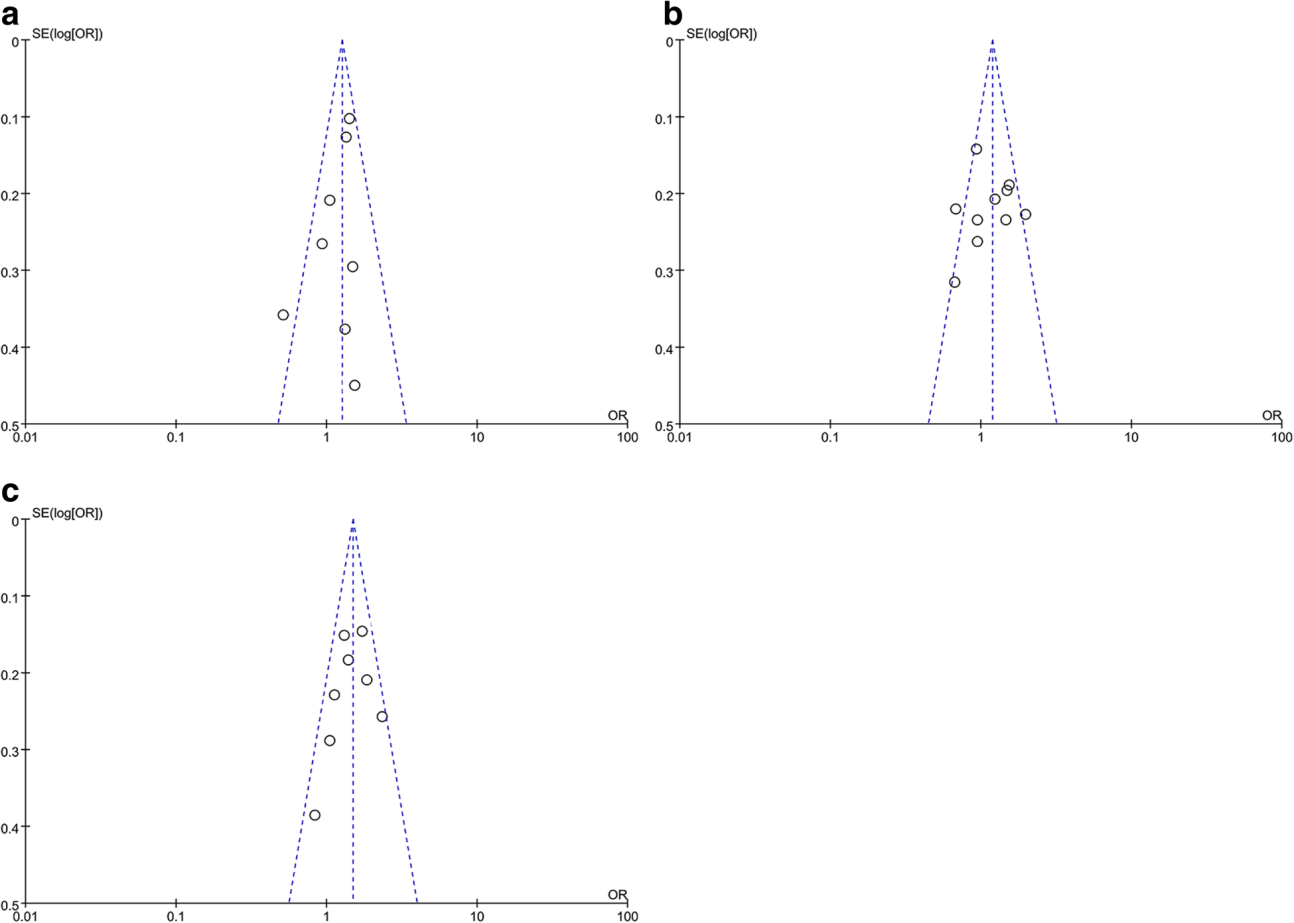
Funnel plot for studies investigating the effect of VEGF gene polymorphisms on CAD risk. **a** Funnel plot for publication bias on the rs699947 polymorphism; **b** Funnel plot for publication bias on the rs2010963 polymorphism; **c** Funnel plot for publication bias on the rs3025039 polymorphism. Abbreviation: OR = odds ratio

We performed funnel plots and Egger’s test to assess the publication bias of the included studies. A funnel plot was not constructed for the 3 rs1570360 studies. The funnel plot distribution of distinct studies appeared nearly symmetrical (Fig. 3). Moreover, Egger’s test failed to show statistically significant asymmetry in dominant genetic models (rs699947: *t* = − 1.21, *P* = 0.270; rs2010963: *t* = − 0.62, *P* = 0.551; rs3025039: *t* = − 2.07, *P* = 0.086; rs1570360: *t* = − 3.05, *P* = 0.787).

## Discussion

VEGFs have been reported to alleviate complications closely linked to CAD by promoting the recanalization of thrombus-blocked blood vessels, establishing collateral circulation against myocardial ischaemia, and improving endothelium-dependent vasodilatation [9, 34, 35]. However, despite the important impacts of hereditary factors on CAD development, a link between VEGF SNPs and CAD risk has yet to be sufficiently elucidated.

In this meta–analysis, we analysed 29 eligible studies and found that rs699947, rs2010963, and rs3025020 polymorphisms increased CAD susceptibility, suggesting that these polymorphisms may be risk factors for CAD. However, the rs1570360 polymorphism failed to yield an association with CAD. One possible explanation for this finding is that the functional polymorphisms of rs699947, rs2010963, and rs3025020 may have more profound effects on angiogenesis than other SNPs. This discrepancy may also result in inter-individual differences in CAD incidence. The findings reported here are in agreement with some studies [21, 28] but not others [23]. The estimated pooled OR did not obviously change when non-HWE studies were excluded, suggesting the stability of the results. Subgroup analysis based on ethnicity identified an association between VEGF gene (rs699947 and rs3025039) polymorphisms and CAD, especially in Asian populations. This discrepancy may be due to genetic heterogeneity among different ethnicities. Moreover, to relieve heterogeneity bias within Asian and small sample size subgroups, stratified analysis was performed and suggested that ethnicity and sample size may be potential sources of heterogeneity. In addition, NOS quality assessment showed no obvious publication bias in our study, which supports the reliability of the conclusions.

Whether VEGF is a pro–atherosclerotic or anti–atherosclerotic factor is currently under debate. Some studies have reported that VEGF plays a role in blood vessel growth and the regulation of vessel wall integrity by promoting regeneration of endothelial cells, enhancing endothelial function, and slowing smooth muscle differentiation [34, 36, 37]. Grosskreutz et al. [38] observed that VEGF expression accelerates re-endothelialisation and reduces intimal thickening and thrombus formation. Howell et al. [39] also reported that higher VEGF expression has a protective effect in atherosclerosis development. In contrast, other studies have shown that VEGF induces atherosclerosis via promotion of vascular smooth muscle cell proliferation and plaque neovascularization [35, 40]. Eaton et al. [41] reported that elevated levels of VEGF were significantly associated with CAD mortality after 13 years of follow–up. Additionally, recent evidence has suggested that low VEGF levels may be needed to maintain vascular homeostasis, whereas upregulated VEGF has been observed in active angiogenesis processes during acute or stable ischaemic myocardium [42–44]. Overall, these data suggest that changes in VEGF levels may reflect the progressive stages of angiogenesis activity.

Several association studies have reported that the + 405 C/G, +936C/T, and − 2578 A/C polymorphisms have an impact on VEGF protein synthesis [12, 39, 45]. However, several functional SNPs in the VEGF gene may contribute to CAD development, possibly by altering VEGF expression or protein activity. It is important to note that several stimulatory factors associated with hypoxia, oxidative stress, hyperglycaemia, hormones, and cytokines can influence plasma VEGF levels [9, 24, 46]. Furthermore, since –2578A/C is in strong linkage disequilibrium with –1154G/A, carriers of the risk allele − 2578 C will in most cases also be carriers of the − 1154 An allele, which appears to lead to enhanced VEGF expression [47]. Based on the above analysis, we speculate that variations in the VEGF gene may be susceptibility factors and outcome predictors for CAD.

Several limitations in our meta–analysis should be addressed. First, heterogeneity may have influenced the interpretation of our results. The limited number of studies with small sample sizes may have influenced the reliability of the conclusions; however, the heterogeneity was reduced by the subgroup analysis. Moreover, further relevant studies may supplement the present conclusions. Second, because of the limitation of the available data that were extracted from each selected study, our results were based on unadjusted estimates, which may produce misleading results. Third, despite searching comprehensive databases, only Asian and Caucasian populations were included in our meta–analysis. Ethnicity bias may exist in our analysis, and the conclusions may not be applicable to other races. Fourth, in our meta-analysis, the case group of several eligible studies consisted of patients with MI only. Most MIs occur due to CAD, despite the pathophysiology of MI is partly different from that of CAD. This discrepancy may increase the clinical heterogeneity among the studies.

## Conclusions

In conclusion, the current meta-analysis supports the existence of an association between VEGF gene polymorphisms (rs699947, rs2010963, and rs3025039) and susceptibility to CAD, especially in Asian populations. Although a subgroup analysis was used to investigate the source of the heterogeneity, the results should be interpreted with caution.

## Additional file

Additional file 1: **Table S1.** MOOSE checklist for meta-analysis. **Table S2.** Methodological quality of the included studies according to the Newcastle-Ottawa Scale. **Figure S1.** Forest plot for the association between the VEGF rs699947 polymorphism and CAD risk in dominant genetic models. **Figure S2.** Forest plot for the association between the VEGF rs699947 polymorphism and CAD risk in recessive genetic models. **Figure S3.** Forest plot for the association between the VEGF rs699947 polymorphism and CAD risk in heterozygous genetic models. **Figure S4.** Forest plot for the association between the VEGF rs699947 polymorphism and CAD risk in homozygous genetic models. **Figure S5.** Forest plot for the association between the VEGF rs699947 polymorphism and CAD risk in allele genetic models. **Figure S6.** Forest plot for the association between the VEGF rs2010963 polymorphism and CAD risk in dominant genetic models. **Figure S7.** Forest plot for the association between the VEGF rs2010963 polymorphism and CAD risk in recessive genetic models. **Figure S8.** Forest plot for the association between the VEGF rs2010963 polymorphism and CAD risk in heterozygous genetic models. **Figure S9.** Forest plot for the association between the VEGF rs2010963 polymorphism and CAD risk in homozygous genetic models. **Figure S10.** Forest plot for the association between the VEGF rs2010963 polymorphism and CAD risk in allele genetic models. **Figure S11.** Forest plot for the association between the VEGF rs3025039 polymorphism and CAD risk in dominant genetic models. **Figure S12.** Forest plot for the association between the VEGF rs3025039 polymorphism and CAD risk in recessive genetic models. **Figure S13.** Forest plot for the association between the VEGF rs3025039 polymorphism and CAD risk in heterozygous genetic models. **Figure S14.** Forest plot for the association between the VEGF rs3025039 polymorphism and CAD risk in homozygous genetic models. **Figure S15.** Forest plot for the association between the VEGF rs3025039 polymorphism and CAD risk in allele genetic models. (DOC 3267 kb)

## Abbreviations

CAD: Coronary artery disease
CIs: Confidence intervals
HWE: Hardy–Weinberg equilibrium
MI: Myocardial infarction
MOOSE: Meta–analysis of Observational Studies in Epidemiology
NOS: Newcastle–Ottawa Scale
ORs: Odds ratios
SNPs: Single nucleotide polymorphisms
VEGF: Vascular endothelial growth factor
