# Supplementary material for: Association of genetic polymorphisms in vascular endothelial growth factor with susceptibility to coronary artery disease: a meta–analysis
Source: BMC Med Genet. 2018 Jul 4;19:108. doi: 10.1186/s12881-018-0628-3 (PMC6031176; doi:10.1186/s12881-018-0628-3)

**Additional file 1**

**Table S1. MOOSE checklist for meta-analysis of observational studies.**

**Table S2. Methodological quality of the included studies according to the Newcastle-Ottawa Scale.**

**Figure S1. Forest plot of odds ratios for the association between the VEGF rs699947 polymorphism and CAD risk in dominant genetic models.**

**Figure S2. Forest plot of odds ratios for the association between the VEGF rs699947 polymorphism and CAD risk in recessive genetic models.**

**Figure S3. Forest plot of odds ratios for the association between the VEGF rs699947 polymorphism and CAD risk in heterozygous genetic models.**

**Figure S4. Forest plot of odds ratios for the association between the VEGF rs699947 polymorphism and CAD risk in homozygous genetic models.**

**Figure S5. Forest plot of odds ratios for the association between the VEGF rs699947 polymorphism and CAD risk in allele genetic models.**

**Figure S6. Forest plot of odds ratios for the association between the VEGF rs2010963 polymorphism and CAD risk in dominant genetic models.**

**Figure S7. Forest plot of odds ratios for the association between the VEGF rs2010963 polymorphism and CAD risk in recessive genetic models.**

**Figure S8. Forest plot of odds ratios for the association between the VEGF rs2010963 polymorphism and CAD risk in heterozygous genetic models.**

**Figure S9. Forest plot of odds ratios for the association between the VEGF rs2010963 polymorphism and CAD risk in homozygous genetic models.**

**Figure S10. Forest plot of odds ratios for the association between the VEGF rs2010963 polymorphism and CAD risk in allele genetic models.**

**Figure S11. Forest plot of odds ratios for the association between the VEGF rs3025039** **polymorphism and CAD risk in dominant genetic models.**

**Figure S12. Forest plot of odds ratios for the association between the VEGF rs3025039** **polymorphism and CAD risk in recessive genetic models.**

**Figure S13. Forest plot of odds ratios for the association between the VEGF rs3025039** **polymorphism and CAD risk in heterozygous genetic models.**

**Figure S14. Forest plot of odds ratios for the association between the VEGF rs3025039** **polymorphism and CAD risk in homozygous genetic models.**

**Figure S15. Forest plot of odds ratios for the association between the VEGF rs3025039** **polymorphism and CAD risk in allele genetic models.**

**Table S1. MOOSE checklist for meta-analysis of observational studies.**

| **Item No** | **Recommendation** | **Reported on Page No** |
| --- | --- | --- |
| Reporting of background should include | | |
| 1 | Problem definition | 2-3 |
| 2 | Hypothesis statement | 2-3 |
| 3 | Description of study outcome(s) | 2-3 |
| 4 | Type of exposure or intervention used | 2-3 |
| 5 | Type of study designs used | 2-3 |
| 6 | Study population | 2-3 |
| Reporting of search strategy should include | | |
| 7 | Qualifications of searchers (eg, librarians and investigators) | 4 |
| 8 | Search strategy, including time period included in the synthesis and key words | 4 |
| 9 | Effort to include all available studies, including contact with authors | 4 |
| 10 | Databases and registries searched | 4 |
| 11 | Search software used, name and version, including special features used (eg, explosion) | 4 |
| 12 | Use of hand searching (eg, reference lists of obtained articles) | 4 |
| 13 | List of citations located and those excluded, including justification | 6-7 |
| 14 | Method of addressing articles published in languages other than English | 4 |
| 15 | Method of handling abstracts and unpublished studies | 4 |
| 16 | Description of any contact with authors | No |
| Reporting of methods should include | | |
| 17 | Description of relevance or appropriateness of studies assembled for assessing the hypothesis to be tested | 4-6 |
| 18 | Rationale for the selection and coding of data (eg, sound clinical principles or convenience) | 4-6 |
| 19 | Documentation of how data were classified and coded (eg, multiple raters, blinding and interrater reliability) | 4-5 |
| 20 | Assessment of confounding (eg, comparability of cases and controls in studies where appropriate) | 5-6 |
| 21 | Assessment of study quality, including blinding of quality assessors, stratification or regression on possible predictors of study results | 5 |
| 22 | Assessment of heterogeneity | 5-6 |
| 23 | Description of statistical methods (eg, complete description of fixed or random effects models, justification of whether the chosen models account for predictors of study results, dose-response models, or cumulative meta-analysis) in sufficient detail to be replicated | 5-6 |
| 24 | Provision of appropriate tables and graphics | 5-6 |
| Reporting of results should include | | |
| 25 | Graphic summarizing individual study estimates and overall estimate | 6-14 |
| 26 | Table giving descriptive information for each study included | 6-14 |
| 27 | Results of sensitivity testing (eg, subgroup analysis) | 7-14 |
| 28 | Indication of statistical uncertainty of findings | 14-16 |
| **Item No** | **Recommendation** | **Reported on Page No** |
| Reporting of discussion should include | | |
| 29 | Quantitative assessment of bias (eg, publication bias) | 17-18 |
| 30 | Justification for exclusion (eg, exclusion of non-English language citations) | 17-18 |
| 31 | Assessment of quality of included studies | 17-18 |
| Reporting of conclusions should include | | |
| 32 | Consideration of alternative explanations for observed results | 17 |
| 33 | Generalization of the conclusions (ie, appropriate for the data presented and within the domain of the literature review) | 16-18 |
| 34 | Guidelines for future research | 18 |
| 35 | Disclosure of funding source | No |

**Table S2**. Methodological quality of the included studies according to the Newcastle-Ottawa Scale.

| Study | Adequacy of  Case Definition | | | Representativeness  of the Cases | Selection of  Controls | | | Definition  of Controls | | Comparability  Cases/Controls | | Ascertainment  of Exposure | | Same Method of  Ascertainment | Non-response  rate | | |
| --- | --- | --- | --- | --- | --- | --- | --- | --- | --- | --- | --- | --- | --- | --- | --- | --- | --- |
| **rs699947** | |  |  | | |  |  | |  | |  | |  | | |  |  |
| Biselli 2008 | | * | * | | | NA | * | | * | | * | | * | | | NA |  |
| Kangas-Kontio 2009 | | * | * | | | NA | * | | ** | | * | | * | | | NA |  |
| Chen 2011 | | * | * | | | * | * | | ** | | * | | * | | | NA |  |
| Amoli 2012 | | * | * | | | NA | * | | * | | * | | * | | | NA |  |
| Cui 2013 | | * | * | | | NA | * | | * | | * | | * | | | NA |  |
| Gu 2013 | | * | * | | | NA | * | | * | | * | | * | | | NA |  |
| Li 2016 | | * | * | | | * | * | | ** | | * | | * | | | NA |  |
| Liu 2016 | | * | * | | | * | * | | ** | | * | | * | | | NA |  |
| **rs2010963** | |  |  | | |  |  | |  | |  | |  | | |  |  |
| Petrovic 2007 | | * | * | | | * | * | | * | | * | | * | | | NA |  |
| Kangas-Kontio 2009 | | * | * | | | NA | * | | ** | | * | | * | | | NA |  |
| Chen 2011 | | * | * | | | * | * | | ** | | * | | * | | | NA |  |
| Cui 2013 | | * | * | | | NA | * | | * | | * | | * | | | NA |  |
| Gu 2013 | | * | * | | | NA | * | | * | | * | | * | | | NA |  |
| Moradzadegan 2015 | | * | * | | | NA | * | | ** | | * | | * | | | NA |  |
| Han 2015 | | * | * | | | NA | * | | * | | * | | * | | | NA |  |
| Li 2016 | | * | * | | | * | * | | ** | | * | | * | | | NA |  |
| **rs3025039** | |  |  | | |  |  | |  | |  | |  | | |  |  |
| Biselli 2008 | | * | * | | | NA | * | | * | | * | | * | | | NA |  |
| Kangas-Kontio 2009 | | * | * | | | NA | * | | ** | | * | | * | | | NA |  |
| Chen 2011 | | * | * | | | * | * | | ** | | * | | * | | | NA |  |
| Cui 2013 | | * | * | | | NA | * | | * | | * | | * | | | NA |  |
| Gu 2013 | | * | * | | | NA | * | | * | | * | | * | | | NA |  |
| Han 2015 | | * | * | | | NA | * | | * | | * | | * | | | NA |  |
| Liu 2016 | | * | * | | | * | * | | ** | | * | | * | | | NA |  |
| **rs1570360** | |  |  | | |  |  | |  | |  | |  | | |  |  |
| Biselli 2008 | | * | * | | | NA | * | | * | | * | | * | | | NA |  |
| Cui 2013 | | * | * | | | NA | * | | * | | * | | * | | | NA |  |
| Liu 2016 | | * | * | | | * | * | | ** | | * | | * | | | NA |  |

This table identifies ‘high’ quality choices with a ‘star’. A study can be awarded a maximum of 1 star for each numbered item within the Selection and Exposure categories. A maximum of 2 stars can be given for Comparability. *, Yes; NA, not applicable.

**Figure S1. Forest plot of odds ratios for the association between the VEGF rs699947 polymorphism and CAD risk in dominant genetic models.**


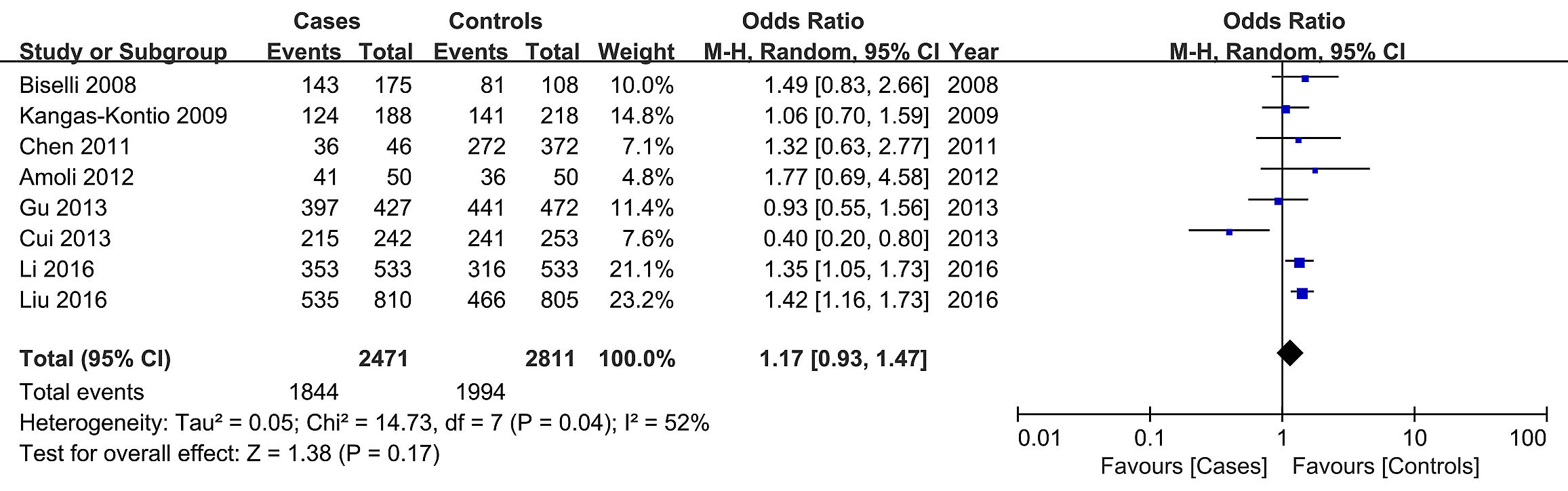


**Figure S2. Forest plot of odds ratios for the association between the VEGF rs699947 polymorphism and CAD risk in recessive genetic models.**


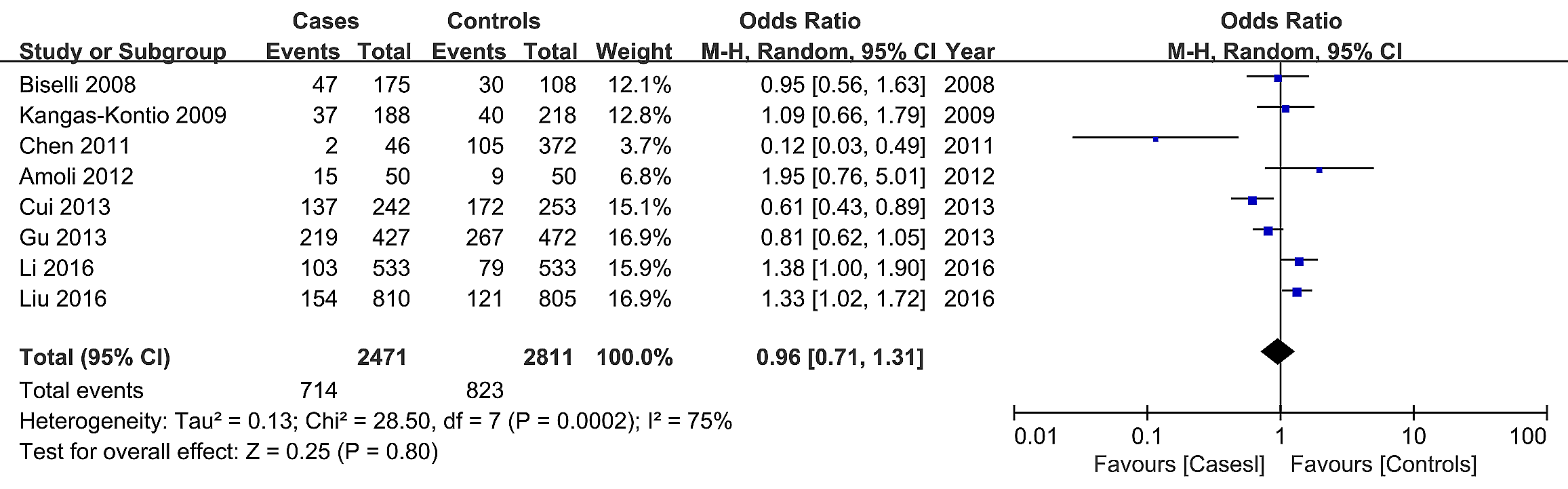


**Figure S3. Forest plot of odds ratios for the association between the VEGF rs699947 polymorphism and CAD risk in heterozygous genetic models.**


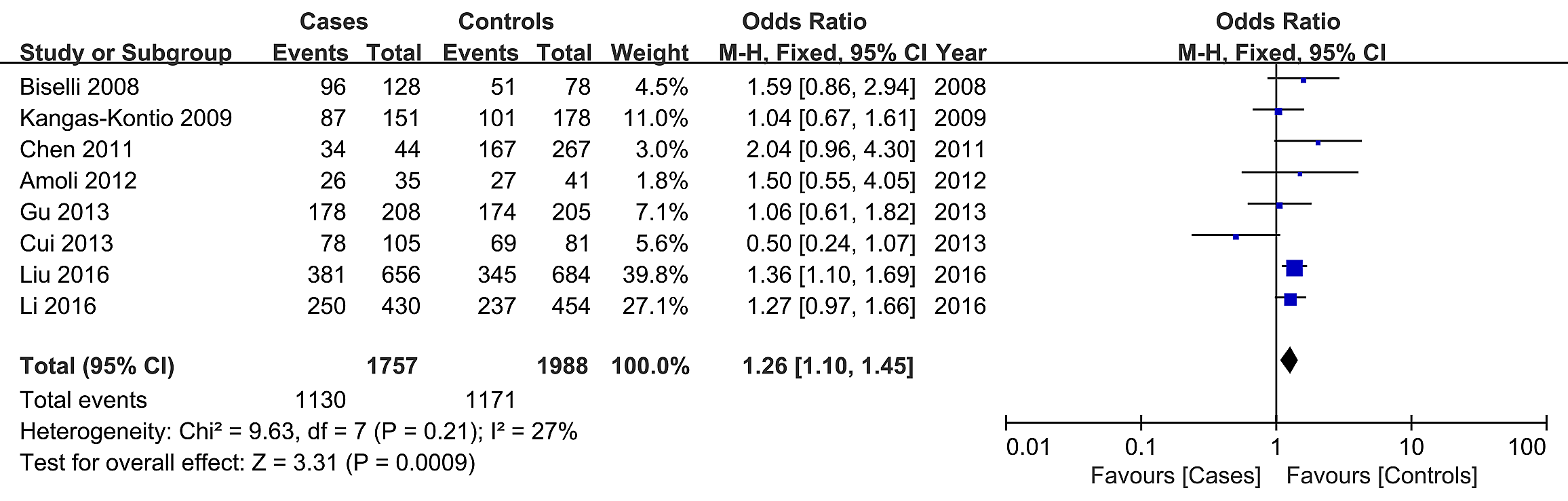


**Figure S4. Forest plot of odds ratios for the association between the VEGF rs699947 polymorphism and CAD risk in homozygous genetic models.**


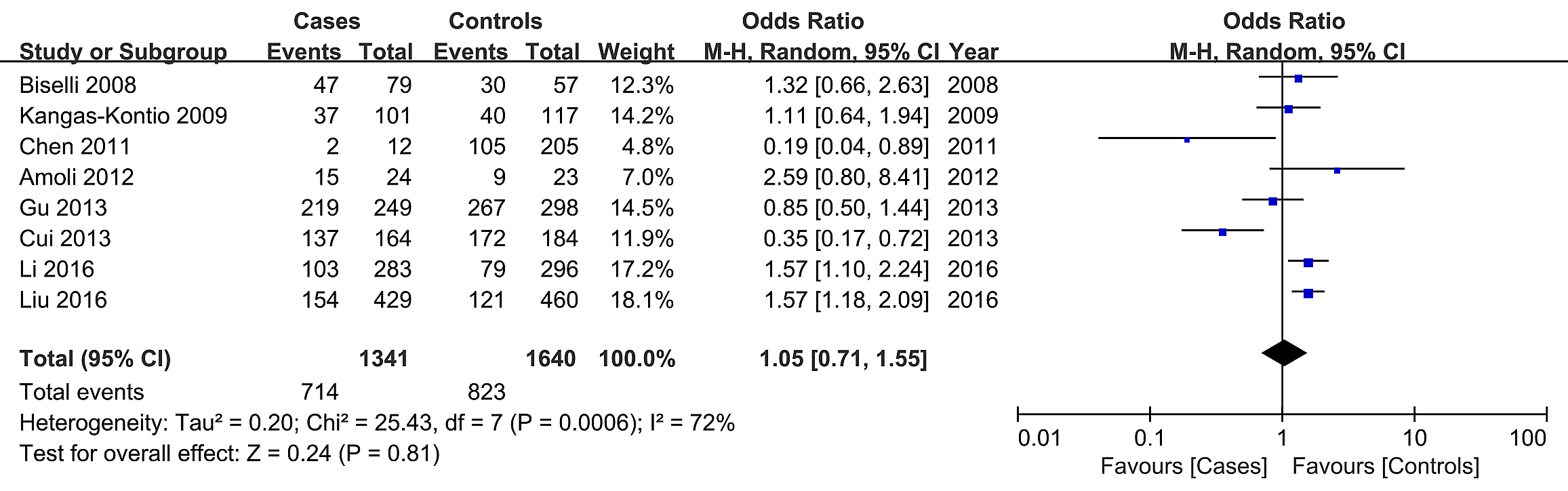


**Figure S5. Forest plot of odds ratios for the association between the VEGF rs699947 polymorphism and CAD risk in allele genetic models.**
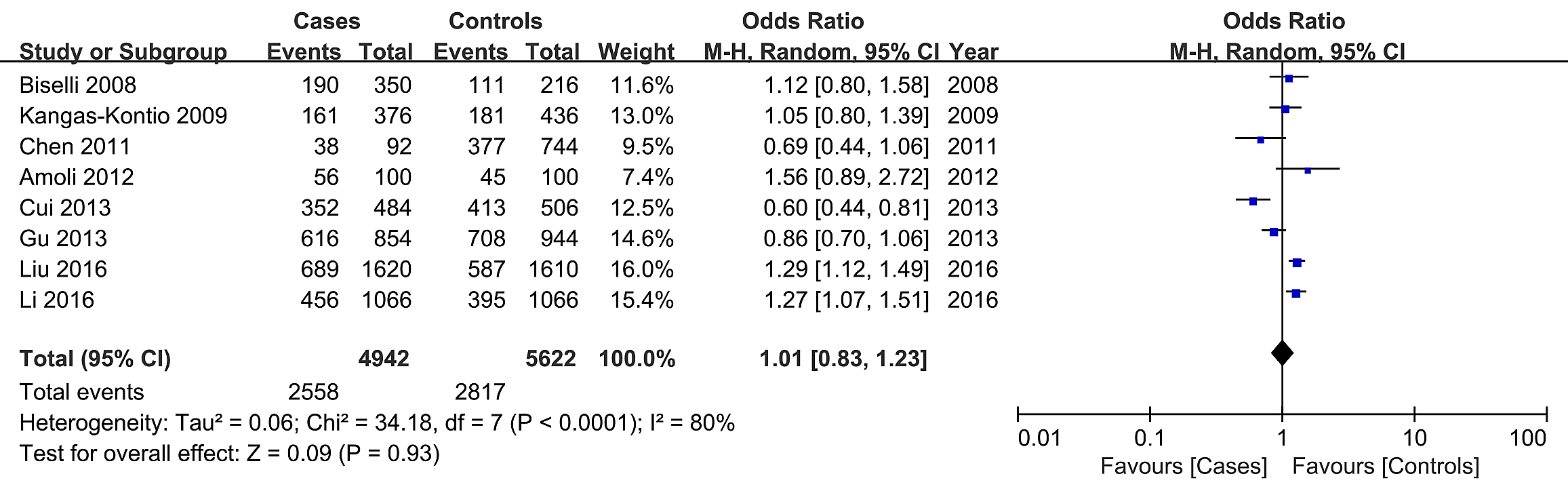


**Figure S6. Forest plot of odds ratios for the association between the VEGF rs2010963 polymorphism and CAD risk in dominant genetic models.**


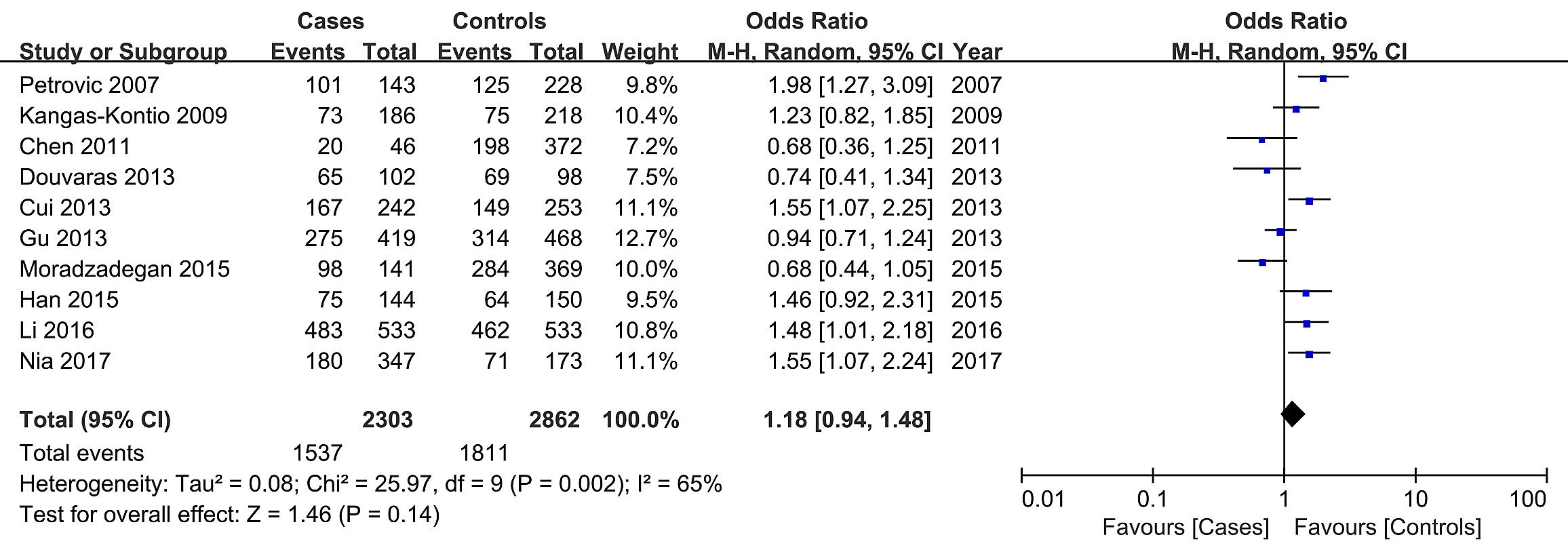


**Figure S7. Forest plot of odds ratios for the association between the VEGF rs2010963 polymorphism and CAD risk in recessive genetic models.**


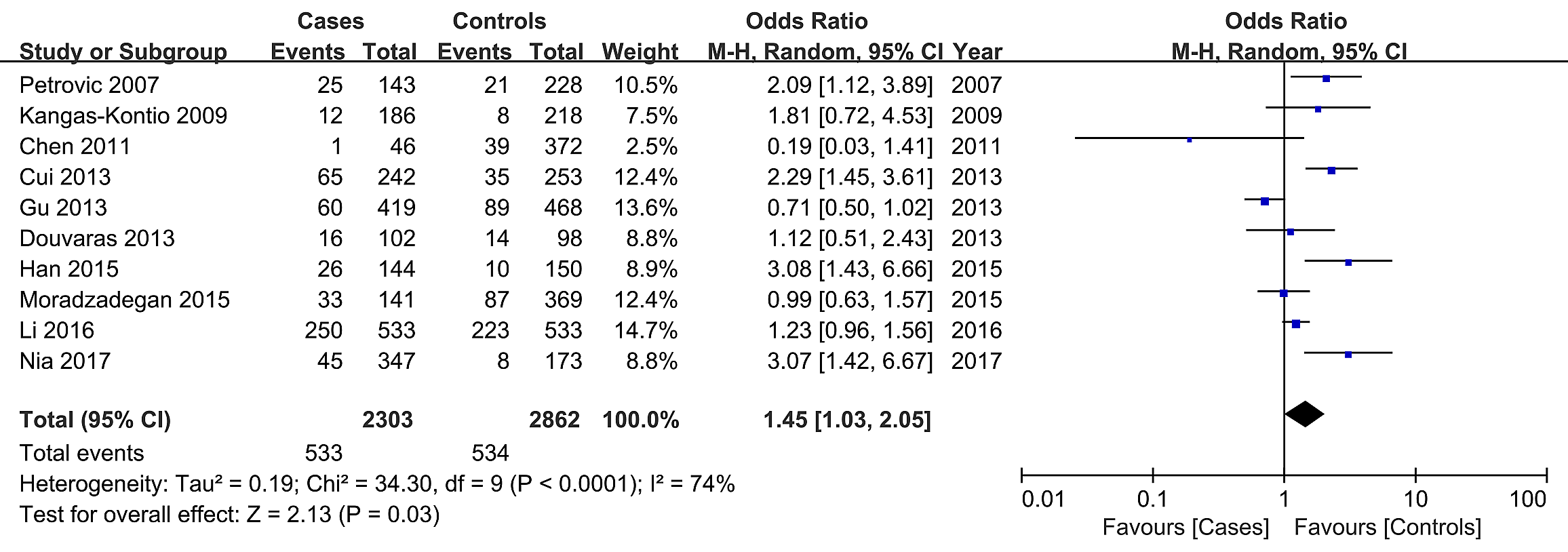


**Figure S8. Forest plot of odds ratios for the association between the VEGF rs2010963 polymorphism and CAD risk in heterozygous genetic models.**


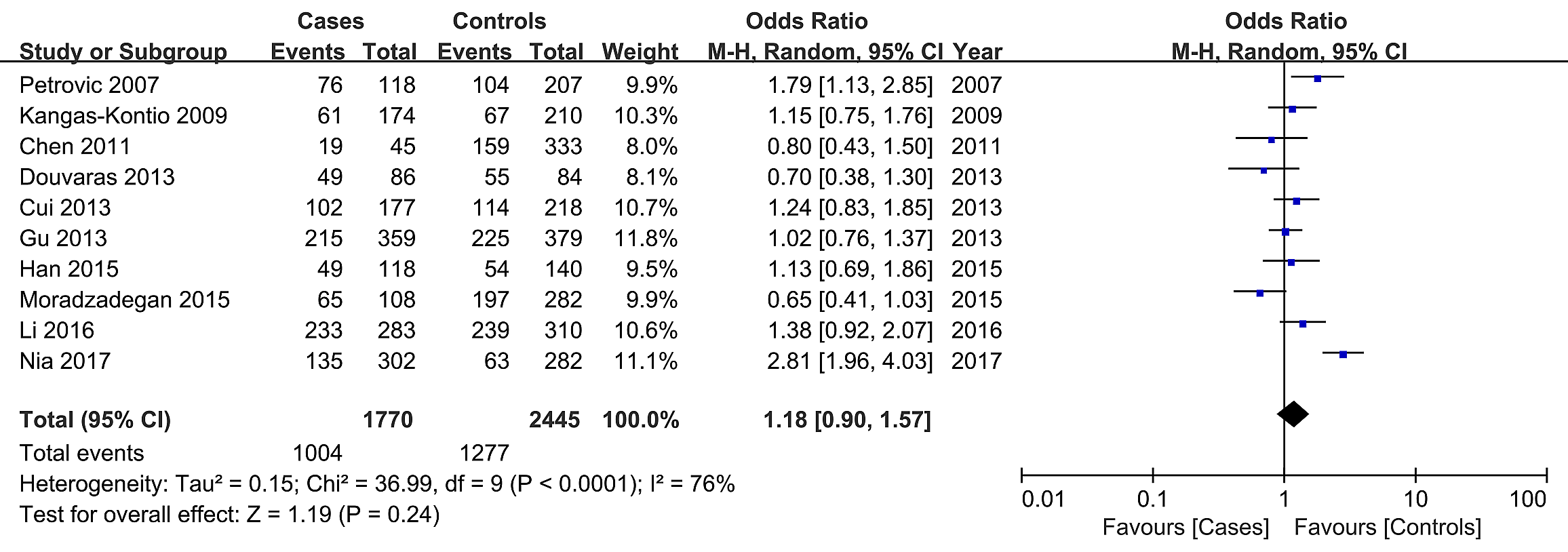


**Figure S9. Forest plot of odds ratios for the association between the VEGF rs2010963 polymorphism and CAD risk in homozygous genetic models.**


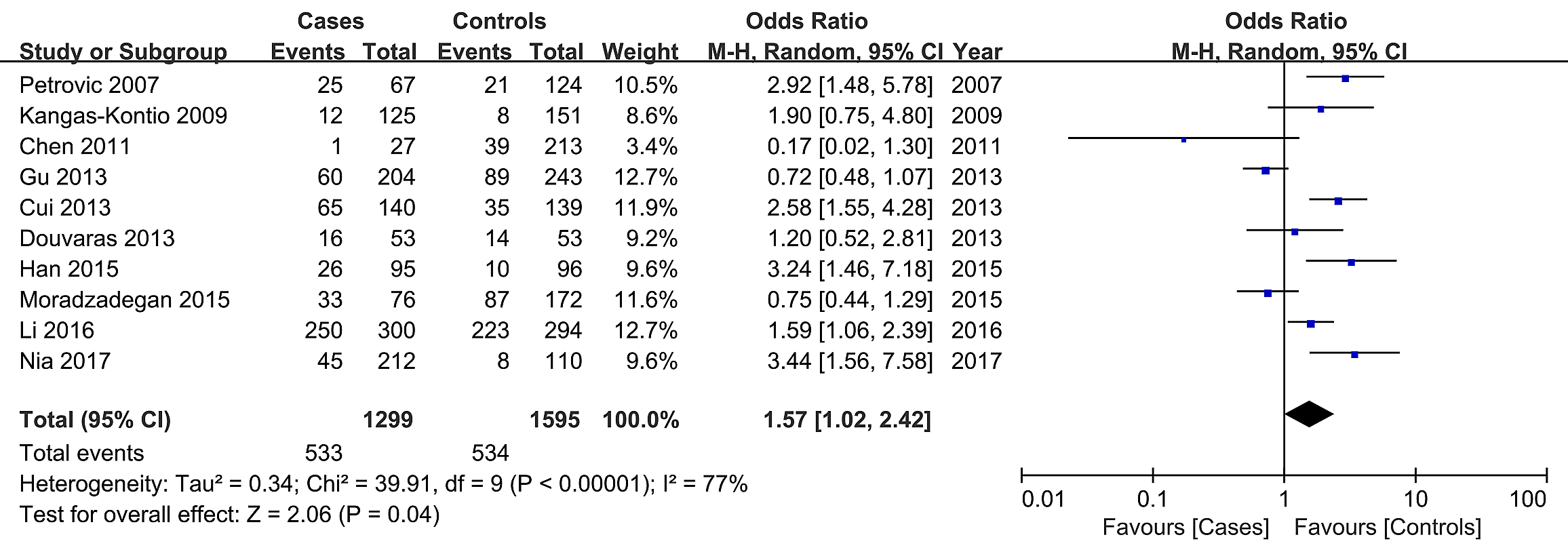


**Figure S10. Forest plot of odds ratios for the association between the VEGF rs2010963 polymorphism and CAD risk in allele genetic models.**


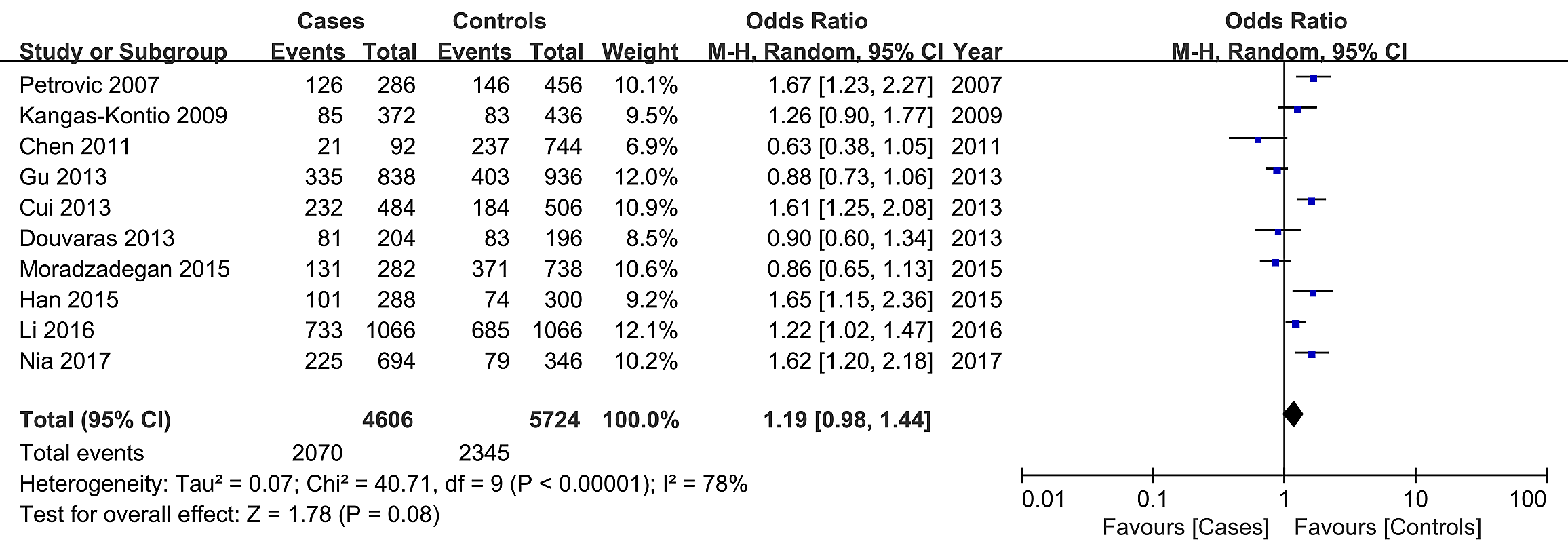


**Figure S11. Forest plot of odds ratios for the association between the VEGF rs3025039** **polymorphism and CAD risk in dominant genetic models.**


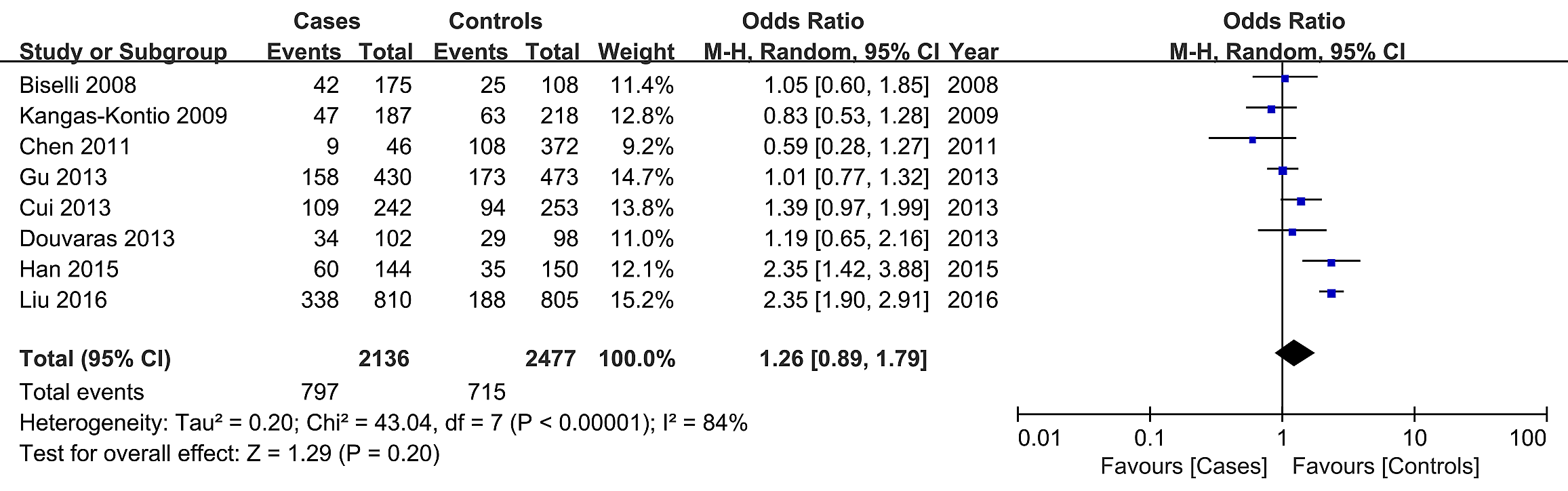


**Figure S12. Forest plot of odds ratios for the association between the VEGF rs3025039** **polymorphism and CAD risk in recessive genetic models.**


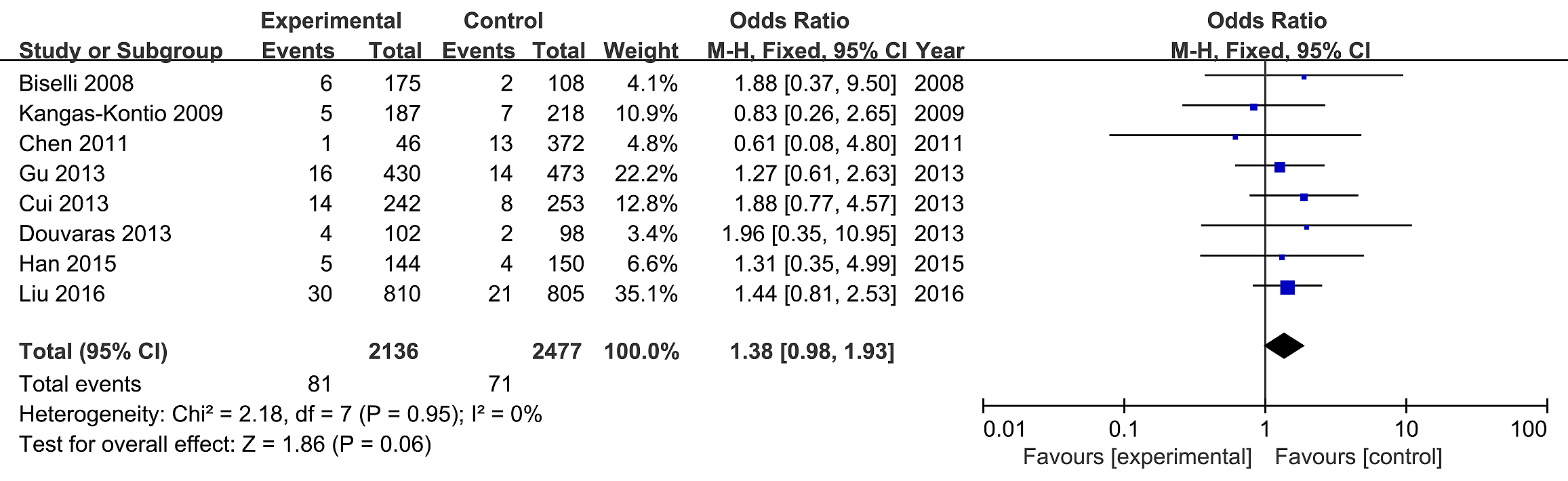


**Figure S13. Forest plot of odds ratios for the association between the VEGF rs3025039** **polymorphism and CAD risk in heterozygous genetic models.**


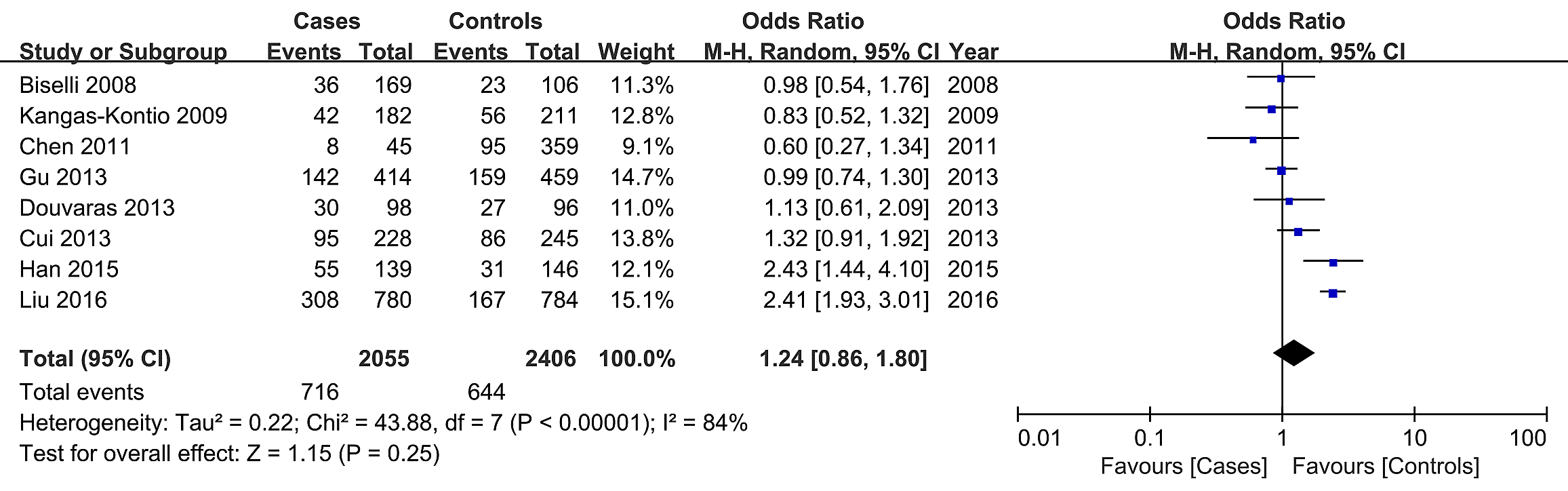


**Figure S14. Forest plot of odds ratios for the association between the VEGF rs3025039** **polymorphism and CAD risk in homozygous genetic models.**


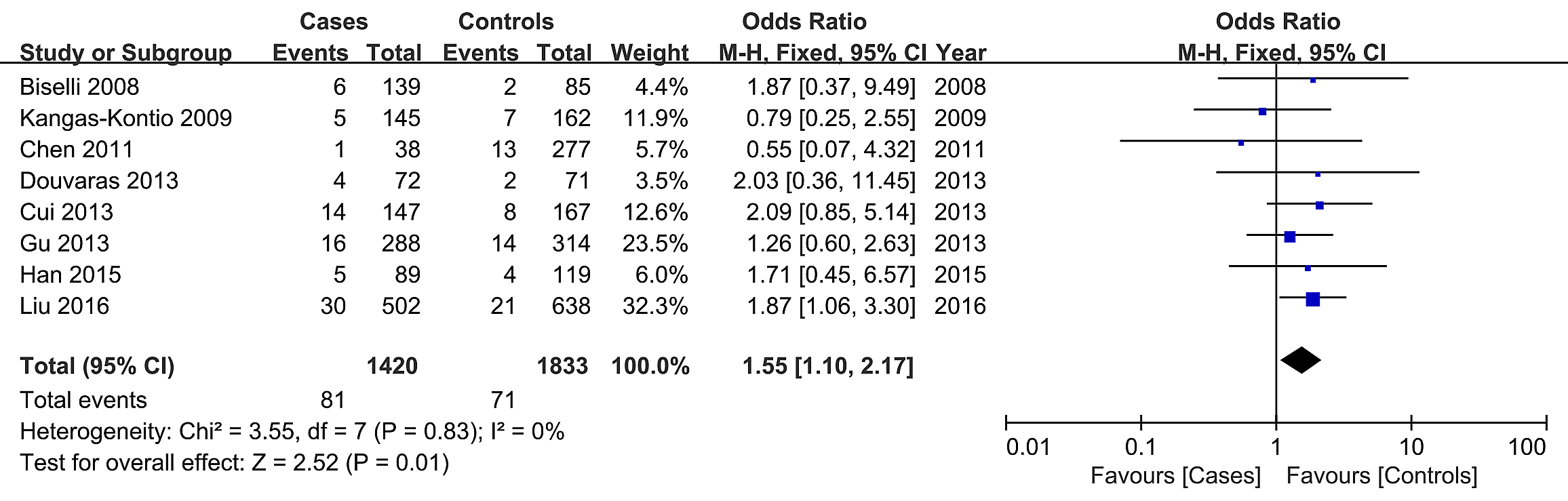


**Figure S15. Forest plot of odds ratios for the association between the VEGF rs3025039** **polymorphism and CAD risk in allele genetic models.**


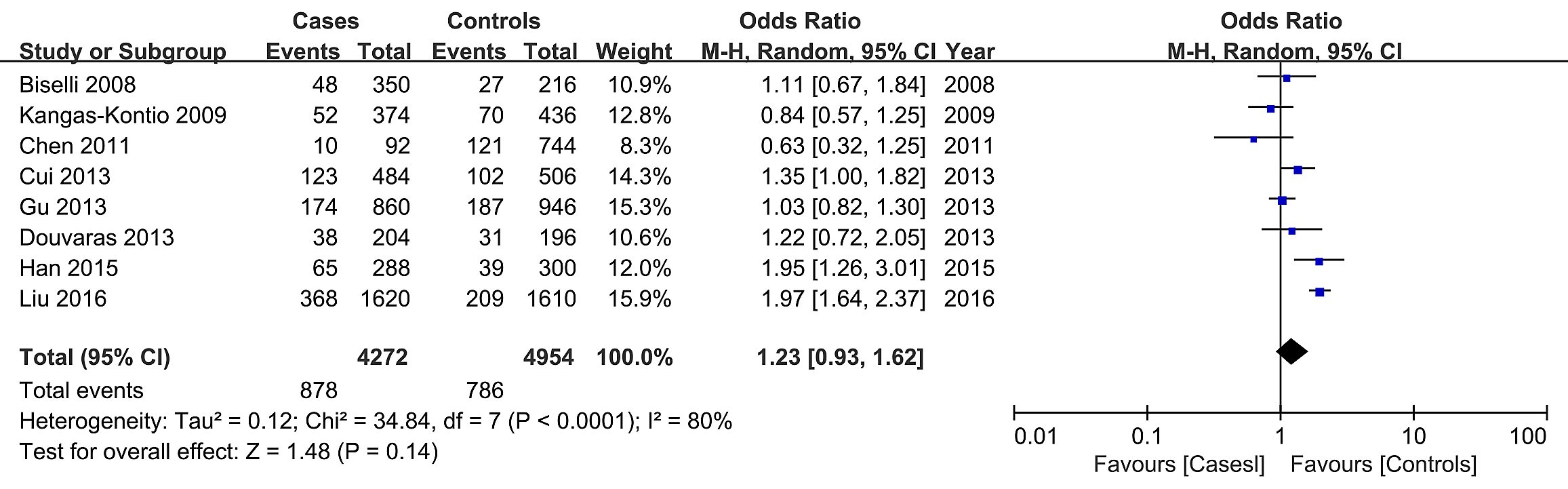

Supplement: Supplementary file 1 — Table S1. MOOSE checklist for meta-analysis. Table S2. Methodological quality of the included studies according to the Newcastle-Ottawa Scale. Figure S1. Forest plot for the association between the VEGF rs699947 polymorphism and CAD risk in dominant genetic models. Figure S2. Forest plot for the association between the VEGF rs699947 polymorphism and CAD risk in recessive genetic models. Figure S3. Forest plot for the association between the VEGF rs699947 polymorphism and CAD risk in heterozygous genetic models. Figure S4. Forest plot for the association between the VEGF rs699947 polymorphism and CAD risk in homozygous genetic models. Figure S5. Forest plot for the association between the VEGF rs699947 polymorphism and CAD risk in allele genetic models. Figure S6. Forest plot for the association between the VEGF rs2010963 polymorphism and CAD risk in dominant genetic models. Figure S7. Forest plot for the association between the VEGF rs2010963 polymorphism and CAD risk in recessive genetic models. Figure S8. Forest plot for the association between the VEGF rs2010963 polymorphism and CAD risk in heterozygous genetic models. Figure S9. Forest plot for the association between the VEGF rs2010963 polymorphism and CAD risk in homozygous genetic models. Figure S10. Forest plot for the association between the VEGF rs2010963 polymorphism and CAD risk in allele genetic models. Figure S11. Forest plot for the association between the VEGF rs3025039 polymorphism and CAD risk in dominant genetic models. Figure S12. Forest plot for the association between the VEGF rs3025039 polymorphism and CAD risk in recessive genetic models. Figure S13. Forest plot for the association between the VEGF rs3025039 polymorphism and CAD risk in heterozygous genetic models. Figure S14. Forest plot for the association between the VEGF rs3025039 polymorphism and CAD risk in homozygous genetic models. Figure S15. Forest plot for the association between the VEGF rs3025039 polymorphism and CAD risk in alle [file 12881_2018_628_MOESM1_ESM.doc]
